# Effect of Vinyl Acetate, Glass Fibers Contents, and Buffer Space on EVA's Mechanical Property and Shock Absorption Ability

**DOI:** 10.1055/s-0044-1779427

**Published:** 2024-05-14

**Authors:** Shinji Togo, Takahiro Sakaue, Arata Tsutsui, Yoshiaki Matsuda, Kazunori Nakajima, Tomotaka Takeda, Kenichi Fukuda, Pekka Vallittu, Lippo Lassila

**Affiliations:** 1 Division of Sports Dentistry, Department of Oral Health and Clinical Science, Tokyo Dental College, Tokyo, Japan; 2 Division of Special Needs Dentistry and Orofacial Pain, Department of Oral Health and Clinical Science, Tokyo Dental College, Tokyo, Japan; 3 Department of Biomaterials Science and TCBC, Institute of Dentistry, University of Turku, Turku, Finland

**Keywords:** sports dentistry, mouthguard material, orofacial injuries, composite materials, materials testing

## Abstract

**Objectives** The aim of the study was to evaluate the mechanical properties and impact absorption capacity of prototype materials comprising ethylene vinyl acetate (EVA) of different hardness reinforced using different amounts of glass fibers (GFs), considering a buffer space.

**Materials and Methods** Six prototype materials were made by adding E-GFs (5 and 10 wt%) to EVA with vinyl acetate (VA) contents of 9.4 wt% (“hard” or HA) and 27.5 wt% (“soft” or SO). Durometer hardness and tensile strength tests were performed to evaluate the mechanical properties of the materials. Moreover, an impact test was conducted using a customized pendulum impact tester to assess the impact absorption capacity (with or without a buffer space) of the specimens.

**Results** The mechanical properties of the prototypes, namely, durometer hardness, Young's modulus, and tensile strength, were significantly higher in the HA group than in the SO group, regardless of the presence or added amount of GFs. The addition of GFs, particularly in a large amount (10 wt%), significantly increased these values. In terms of the impact absorption capacity, the original hardness of the EVA material, that is, its VA content, had a more substantial effect than the presence or absence of GFs and the added amount of GFs. Interestingly, the HA specimens with the buffer space exhibited significantly higher impact absorption capacities than the SO specimens. Meanwhile, the SO specimens without the buffer space exhibited significantly higher impact absorption capacities than the HA specimens. Moreover, regardless of the sample material and impact distance, the buffer space significantly improved impact absorption. In particular, with the buffer space, the impact absorption capacity increased with the added amount of GFs.

**Conclusion** The basic mechanical properties, including durometer hardness, Young's modulus, and tensile strength, of the EVA prototype were significantly increased by reducing the amount of VA regardless of the presence or added amount of GFs. Adding GFs, particularly in large amounts, significantly increased the values of aforementioned mechanical properties. Impact absorption was significantly affected by the hardness of the original EVA material and enhanced by the addition of the buffer space. The HA specimen had a high shock absorption capacity with the buffer space, and the SO specimen had a high shock absorption capacity without the buffer space. With the buffer space, impact absorption improved with the amount of added GFs.

## Introduction

Multiple studies revealed an effect of mouthguards (MGs) on dental trauma. 1 2 3 4 5 In a recent meta-analysis, Fernandes et al indicated MGs' high effectiveness against dental trauma. The prevalence of dentoalveolar trauma among users and nonusers of MGs in high-quality problem-free studies was 8 and 60%, respectively. 5 However, individuals have experienced maxillofacial trauma while wearing MGs. Jagger et al 6 reported that dental injuries are the most prevalent injury among schoolboy rugby players (26% of total dental, orofacial, and head injuries); 11% of their sample had fractured teeth and 4% had avulsed teeth, although all players used MGs at the time of their accidents. Quarrie et al 7 reported that MG wearing is associated with a mere 43% reduction in dental injuries in rugby players from New Zealand. Dorney 8 stated that MGs provide different levels of protection. Injuries occur during the wearing of MGs because of the poor impact absorption capacity of certain MGs.

Increased MG thickness is important as it improves the safety and impact absorption capacity of MGs made of the ethylene vinyl acetate (EVA) copolymer, a polymer that is common in MG applications. 9 10 11 12 However, excessive thickness leads to discomfort during use, and there is a limit to only relying on the thickness of a single material. Therefore, multiple studies have been conducted to improve the impact absorption capacity of MGs. Researchers have attempted to increase safety from the design perspective such as by developing “hard and space” (H&S) MGs; an H&S MG comprises outer and inner EVA layers and a hard-insert middle layer with a buffer space that prevents contact between the inner surfaces of the MG and the labial surface of the maxillary anterior teeth. 13 14 15 16 17 18 Although the effectiveness of this type of MG has been extensively documented, it is difficult to apply to all age groups and event athletes because of its production method and cost issues. Furthermore, researchers have attempted to improve the MG material itself. Westerman et al added air bubbles to an EVA material; their study demonstrated the effectiveness of their method, 19 but their results have not been clinically applied.

EVA copolymers are thermoplastics; they are polyolefins obtained from the random copolymerization of ethylene and vinyl acetate (VA). 20 The mechanical properties of EVA can be controlled in two ways. First, changes in the mixing ratio of ethylene and VA influence EVA's thermal and rheological properties 21 and its mechanical and viscoelastic properties and hardness. 22 23 24 Second, reinforcement materials, such as fibers, can be added; EVA has good compounding properties because of its low crystallinity and can be compounded in multiple ways to suit various purposes. 25

Differences in the VA content of EVA affect various properties of the copolymer. Alothman 20 reported that increasing the VA content of EVA results in a steady decrease in hardness and a rubbery behavior. Therefore, the hardness and overall reduction in mechanical properties of EVA can be attributed to the rubbery nature and low crystallinity of the copolymer because of the high VA content. The mechanical and viscoelastic properties and hardness of EVA decrease because of an increase in VA content. 22 23 24 Such reduced hardness and improved rubberlike properties should enhance the impact absorption capacity of EVA. Furthermore, increasing the VA content significantly reduces EVA crystallinity, 20 23 thus enhancing the loading capacity of additives such as fillers and fibers. 20 22 23 24 25 26 This high loading capacity of EVA is attributed to VA content, which is suitable for compounding.

The addition of a reinforcing material to EVA to develop a composite material can improve the elastic modulus and strength of the copolymer. 27 Fiber-reinforced plastics (FRPs) are composite materials formed using synthetic resins. The reinforcing materials of FRPs include synthetic fibers such as glass, carbon, steel, aramid, nylon, and polyester fibers 28 and natural fibers such as hemp, cotton, and bamboo fibers. Glass fibers (GFs) are popular in denture base materials, and multiple studies on their effects have been published. 29 30 31 32 33 34 35 Therefore, GFs are used to reinforce EVA in this study.

Most MGs are manufactured by thermoforming single sheets of soft materials. Therefore, multiple researchers have examined the impact absorption capacity of MGs composed of soft materials such as EVA. However, MGs in different positions require different material properties. When the inner layer of an MG is made of a hard material, it has been reported that some of the stress generated at the affected area is reduced, which can impede damage to the tooth's surface. 36 Moreover, if EVA comes into contact with each other and it is heated, a strong adhesion can be formed due to their compatibility. Thus, the manufactured EVA materials can be thermoformed. 37 38 39 EVA hardness can be adjusted by varying the amount of VA in the EVA material. Through the hardness, strength, and impact resistance improvement because of the addition of fibers to EVA, it would be possible to produce MGs with the necessary characteristics depending on the different MG parts.

Therefore, this study aims to evaluate the mechanical properties and impact absorption capacity considering a buffer space of prototype materials composed of EVA of different hardness reinforced using different amounts of GFs.

## Materials and Methods

### Fabrication of Prototype EVA, GF, and Materials

Table 1 lists the materials used in this study. In general, the stiffness of an EVA material significantly decreases with an increase in its VA content. Two types of EVA granules were used as polymer matrices, namely, Escorene Ultra FL 00909 (ExxonMobil Chemical, Spring, TX, United States; VA content: 9.4 wt%; named “hard” or HA) and Escorene Ultra UL 02528CC (ExxonMobil Chemical; VA content: 27.5 wt%; named “soft” or SO).

**Table 1:** EVA and fiber used in this study

Material name	Manufacture	Lot no.	Remark	Code
EVA	ExxonMobil Chemical, Spring, TX, United States	909	VA content: 9.4%	HA
	ExxonMobil Chemical, Spring, TX, United States	02528CC	VA content: 27.5%	SO
Glass fiber	GC, Tokyo, Japan	1903271	Average length: 100 μm	GF

Abbreviation: EVA, ethylene vinyl acetate.

Both EVA polymers were reinforced using E-GFs (silane-treated original fibers GFs; GC, Tokyo, Japan). The average length of the original GFs was 100 μm. The fiber-reinforced granules were manufactured via extrusion using a twin screw extruder (LZ-120HP; LabTech Engineering Co. Ltd., Bangpoo Industrial Estate, Samutprakarn, Thailand). The reinforcement materials were manually added to melted EVA during extrusion. The temperature and running parameters were then adjusted as per the EVA grade used. The processing temperatures of the HA and SO EVA were approximately 190 and 150°C, respectively. The reinforced EVA manufactured via extrusion was cut to granules using a cutter (LTE20-44; LabTech Engineering Co. Ltd.). The amounts of added reinforcement fibers were 5 and 10 wt%. The original commercial EVA granules were used as controls. G*Power version 3.1.9.7 was used to perform the priori power analysis. The sample size was determined from the strain at SO0 and SO5. The strains in the preliminary study were SO0 (1,681 ± 13.7) and SO5 (1,675 ± 9.11), calculated with an effect size of 0.52, alpha error of 0.05, and beta error of 0.8, thereby requiring a minimum of 48 impacts.

The reinforced and control EVA granules were pressed to 2-mm-thick disks using a hydraulic press (LP-S-20, LabTech Engineering Co. Ltd.). The pressing temperature was 142°C, and the pressing time for both hard and soft EVA was 10 seconds. Each material was labeled HA0, HA5, HA10, SO0, SO5, and SO10. The disks were then characterized through durometer hardness and tensile strength tests.

**Fig. 1:**
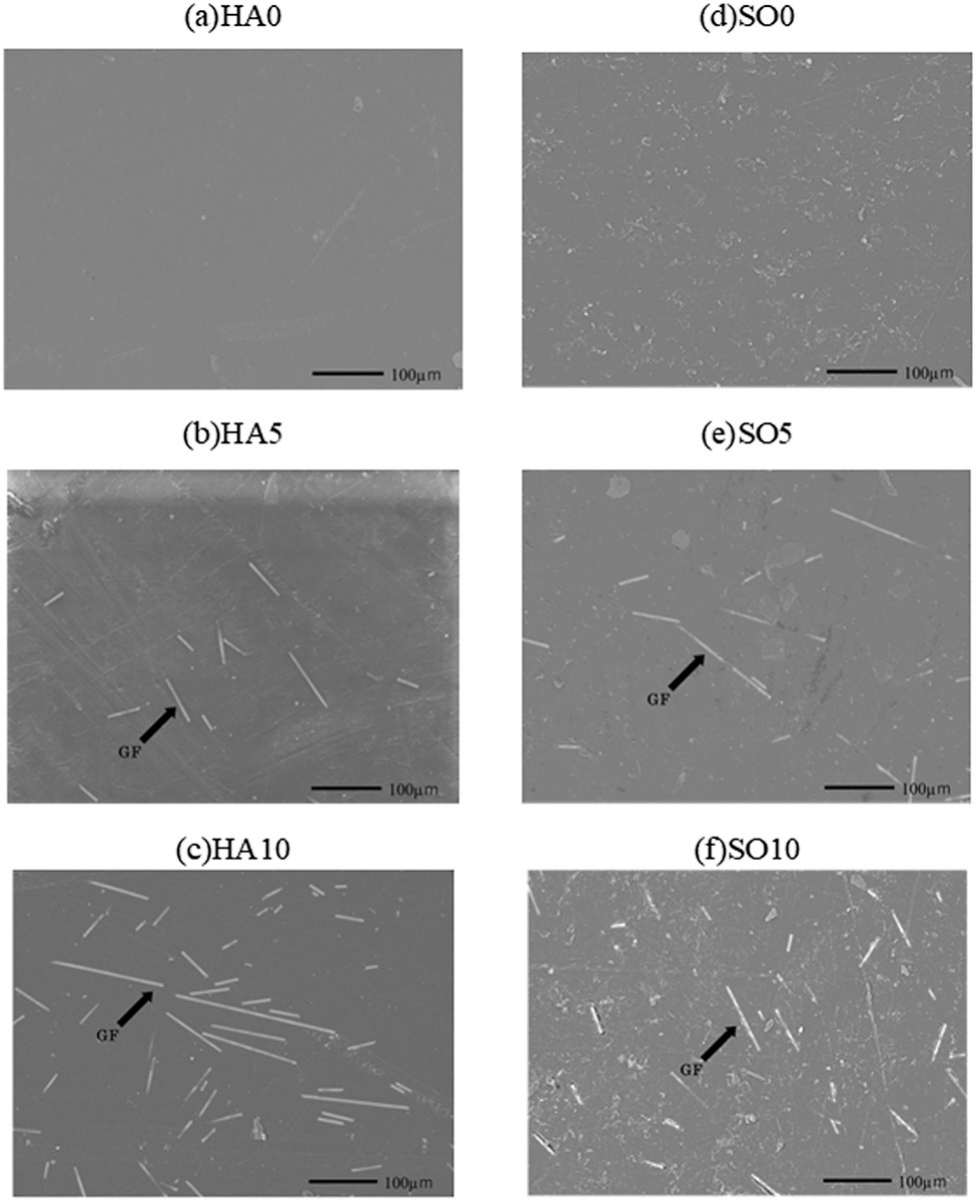
Scanning electron microscopy (SEM) images of specimens. Hard specimens (HA) without glass fibres (GF) ( **A** ). Soft specimens (SO) without GF ( **D** ). In the HA and SO, the GF are shorter or slightly longer than the original average length ( **B, C, E, F** ) ,and the fibers are randomly oriented in the matrices ( **E,F** ). The polymer matrices seemed to be properly impregnated with the fibers ( **B, C, E, F** ).

The prepared specimens were unpolished and deposited with Pt–Platinum-Palladium (Pt-Pd) at a voltage of 15 kV for scanning electron microscopy (SEM) observation ( Fig. 1 ). Fig. 1B, C, E, F shows that the GFs in the HA and SO specimens were shorter or slightly longer than the average length, and the fibers were randomly oriented in the matrices ( Fig. 1B, C, E, F ). The polymer matrices seemed to be properly impregnated with the fibers.

### Durometer Hardness Test

For the durometer hardness test, a 20 mm × 30 mm piece of each disk was used as a test specimen. The durometer hardness was measured using a hardness tester (200 durometer, Shimadzu, Kyoto, Japan). Ten measurements were obtained near the center of each of the five specimens.

### Tensile Test

The tensile strength and Young's modulus were measured from a strip with a width of 10 mm and a length of 60 mm cut out of each disk. The measurement was conducted using a universal testing machine (LR30KPlus 01/3160, 107173, Lloyd Instruments/Ametek, United States) and a 2,500-N load cell. The span length and extension rate were 30 mm and 20 mm/min, respectively; moreover, the test was continued until failure or when the maximum extension of 90 mm was reached. Five test specimens were measured per sample group.

### Impact Shock Absorption Test

**Fig. 2:**
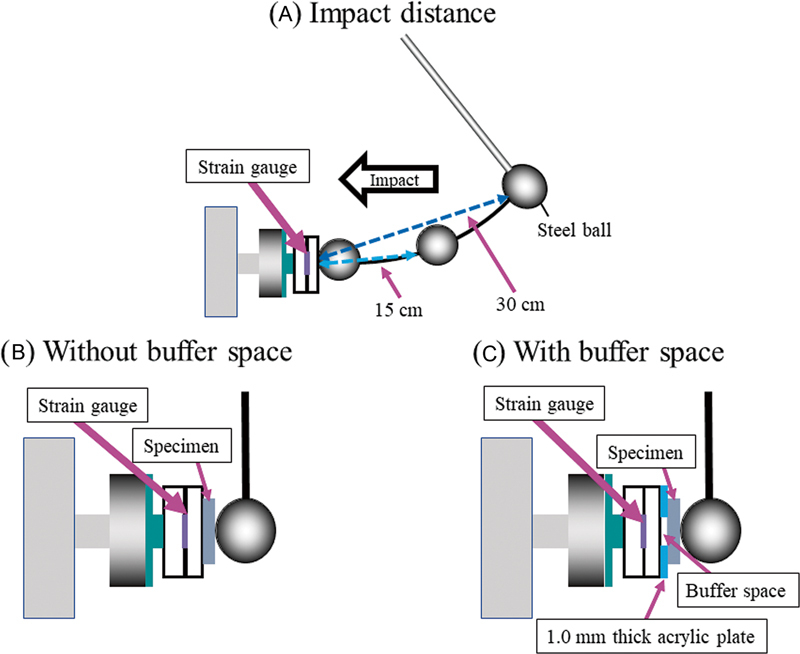
( **A–C** ) Customized pendulum impact testing machine and impact testing conditions.

As per Takeda et al, 15 40 41 42 Matsuda et al, 43 and Sakaue et al, 35 a customized pendulum impact tester with a steel ball (172.5 g in weight, 35 mm in diameter) and acrylic resin plates (5-mm upper plate and 10-mm bottom plate pasted together) with a strain gauge (KFG-1N-120-C1-11L1M2R, Kyowa, Tokyo, Japan) applied to the intermediate layer of the resin plates just below the impact point were used to measure the transmitted strains of the specimens as their impact absorption capacity ( Fig. 2A ). The buffer space was created by pasting a 1-mm-thick acrylic plate ( Fig. 2B ) and a circle with a diameter of 1.5 mm centered on the impact point. The impact point was adjusted using an XYZ-axis rack-and-pinion dovetail stage (TAR-70135, Sigma Koki, Tokyo, Japan) attached to the axial point of the pendulum arm such that the ball could accurately contact the impact point.

The impact distances from the resin plate surface were 15 and 30 cm. 15 40 41 42 43 At each distance without the EVA materials, the impact force was 38 and 68 gf from the accelerometer measurements on the steel ball. The mechanical forces recorded by the strain gauges were amplified and converted into voltage outputs, and the data were stored in a memory recorder/analyzer (EDX-1500A, Kyowa, Tokyo, Japan). The data were then analyzed using a data analysis software (DAS-100A, Kyowa) to calculate the mean value and standard deviation of each variable of strain magnitude at the impact time. The impact absorption capacity of each specimen was calculated based on the mean of the strain values obtained using the following formula:

impact absorption capacity = 100 × (1-each specimen strain/no MG strain) (%).

Five 20 mm × 30 mm with 3-mm-thickness materials were prepared using a pressure molding machine and a funky tool (Drufomat SQ, Dreve Dentamid, Unna, Germany), with the size assuming the anterior tooth area and the thickness having a high trauma prevention effect based on previous studies as a reference. Before the test, the thickness at each impact point was measured using a digital thickness gauge (Model G, Ozaki MFG, Tokyo, Japan) and adjusted to a constant value. Each specimen was then impacted 10 times.

### Statistical Analysis

Statistical analysis was performed using the Statistical Package for the Social Sciences (SPSS) version 25.0 (IBM, Chicago, IL, United States). Normality was confirmed using the Shapiro–Wilk test, and one-way analysis of variance (ANOVA) and the Bonferroni multiple comparison test were performed on the durometer hardness and impact absorption capacity results. In terms of the influence of the presence or absence of the buffer space on the impact absorption capacity, a paired *t* -test was performed between each sample. Kruskal–Wallis and Steel–Dwass multiple comparison tests were performed on Young's modulus and tensile strength test results using BellCurve for Excel (Social Survey Research Information Co. Ltd., Tokyo, Japan). A *p* -value of less than 0.01 was considered significant.

## Results

**Fig. 3:**
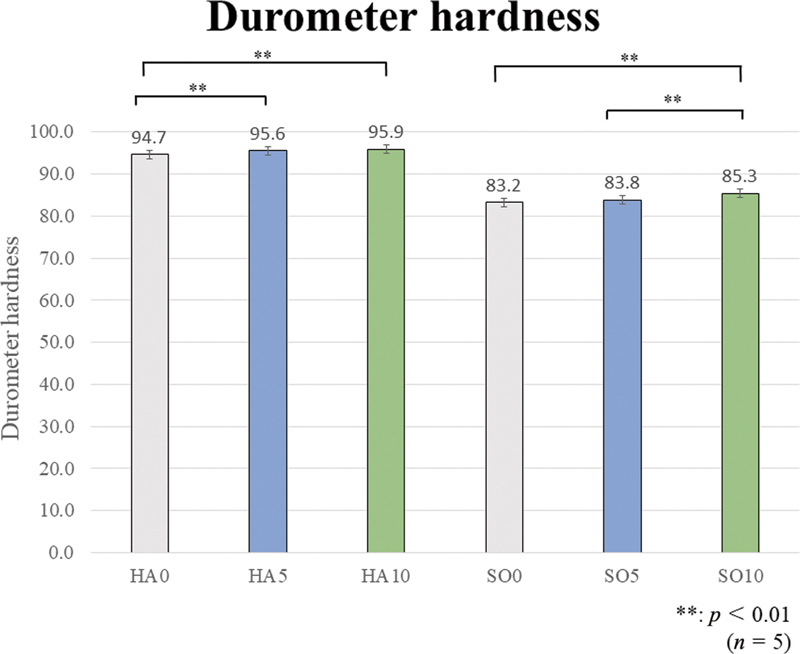
Durometer hardness. The hard (HA) group shows significantly higher values than the soft (SO) group. Regarding the effect of the presence or added amount of glass fibers (GFs), in the HA group, HA10 and HA5 are significantly harder than HA0. In the SO group, SO10 is significantly harder than SO0 and SO5 (** *p* < 0.01).

The one-way ANOVA revealed a significant difference among all prototype materials in the durometer hardness ( Table 2 ). The durometer hardness results are shown in Fig. 3 . The Bonferroni multiple comparison test results are shown in the graph. All group comparison results are significant, but the asterisk symbols (**) indicating significance are not shown in the graph to avoid cluttering the figure.

**Table 2:** Durometer hardness (one-way ANOVA)

	Sum of squares	df	Mean square	*F*	*p*
Factor	9,705.76	5	1,941.152	3,123.898	<0.01
Error	152.24	245	0.621		
Total	9,858	250			

Abbreviation: ANOVA, analysis of variance.

The HA group shows significantly higher values than the SO group. In terms of the effect of the presence and amount of GFs, in the HA group, HA5 (95.6) and HA10 (95.9) were significantly harder than HA0 (94.7). In the SO group, SO10 (85.3) was significantly harder than SO0 (83.2) and SO5 (83.8).

**Fig. 4:**
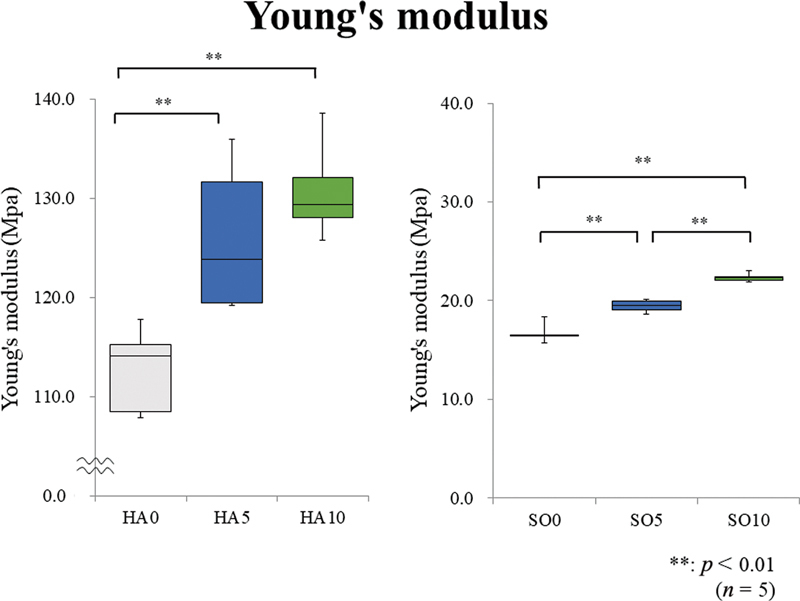
Young's modulus. In the hard (HA) group, HA10 and HA5 show significantly higher values than HA0. In the soft (SO) group, SO10 shows a significantly higher value than SO0 and SO5, and SO5 shows a significantly higher value than SO0.

**Fig. 5:**
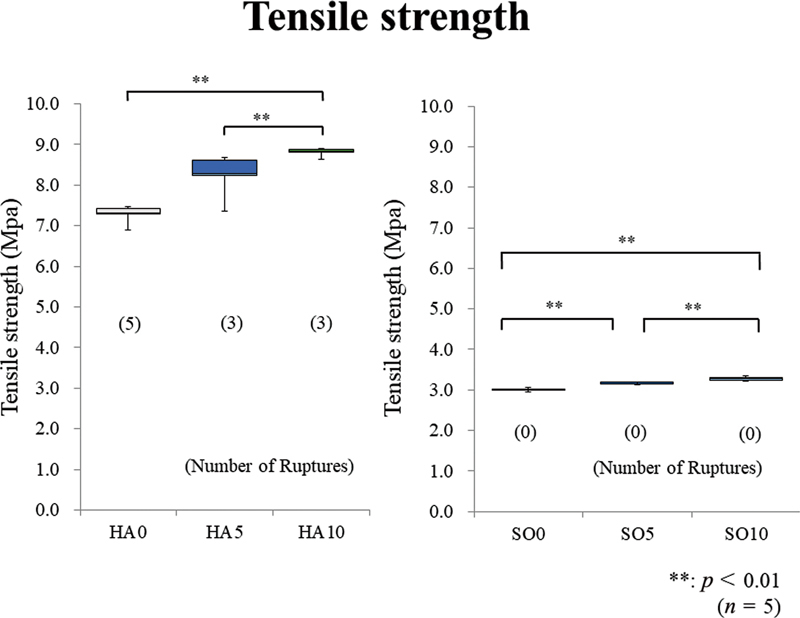
Tensile strength. In the hard (HA) group, HA10 and HA5 show significantly higher values than HA0. In the soft (SO) group, SO10 shows a significantly higher value than SO0 and SO5, and SO5 shows a significantly higher value than SO0. The number of fractured samples also decreases with the added amount of glass fibers (GFs) in the HA group.

The Kruskal–Wallis tests revealed a significant difference among all prototype materials in the tensile tests ( Tables 3 and 4 ). Figs. 4 and 5 show the Young modulus and tensile strength results, respectively. The number of fractured samples is shown in parentheses in Fig. 5 . Moreover, the Steel–Dwass multiple comparison test results are shown in both figures. The multiple comparison tests were separately performed because of the substantial difference between the tensile test results of the HA and SO groups.

**Table 3:** Young's modulus (Kruskal–Wallis test)

	*n*	Mean rank
HA0	5	18.4
HA5	5	22.8
HA10	5	27.8
SO0	5	3
SO5	5	8
SO10	5	13
x2	*F*	*p*
27.8542	5	<0.01**

**: *p* < 0.01.

**Table 4:** Tensile strength (Kruskal–Wallis test)

	*n*	Mean rank
HA0	5	18
HA5	5	24.4
HA10	5	26.6
SO0	5	3
SO5	5	8
SO10	5	13
x2	*F*	*p*
27.5755	5	<0.01**

**: *p* < 0.01.

In the HA group, HA10 and HA5 show significantly higher tensile strength values than HA0. In the SO group, SO10 has a significantly higher value than SO0 and SO5, and SO5 has a significantly higher value than SO0. The number of fractured samples decreases with an increase in the added GF amount in the HA group. Regarding Young's modulus, in the HA group, HA10 and HA5 exhibit significantly higher values than HA0. In the SO group, SO10 shows a significantly higher value than SO0 and SO5, and SO5 has a significantly higher value than SO0.

**Fig. 6:**
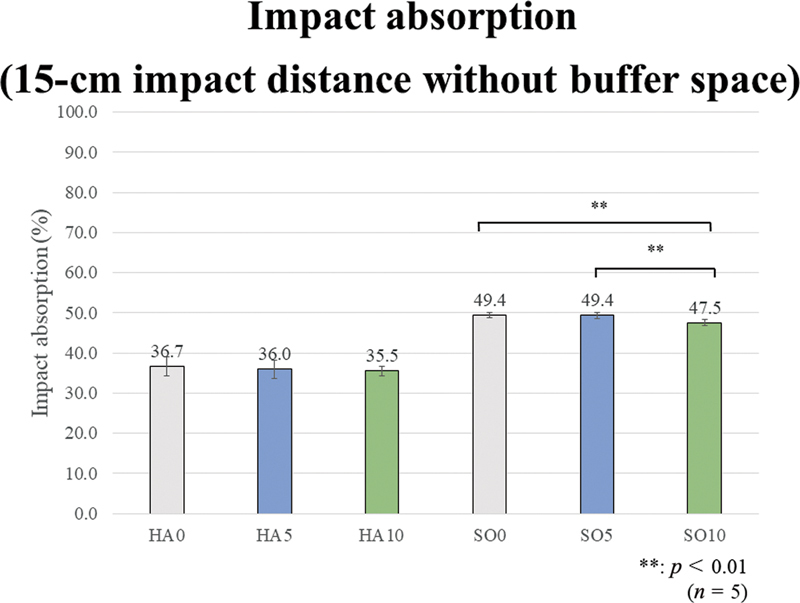
Impact absorption (15-cm impact distance without buffer space). Regarding the effect of the ethylene vinyl acetate (EVA) material hardness, the impact absorption rates of the soft (SO) group are significantly higher than those of the hard (HA) group, regardless of the presence or added amount of glass fibers (GFs). Regarding the effect of fiber addition and content, in the HA group, no significant effect is observed. In the SO group, SO10 shows significantly lower values than SO0 and SO5.

The one-way ANOVA tests revealed a significant difference among all prototype materials in the impact absorption rate under all four test conditions of the impact distances of 15 and 30 cm, without and with buffer space ( Table 5 ). Figs. 6 7 8 9 show the impact absorption rates under each condition against strain without MG, including the Bonferroni multiple comparison test results in each group. All group comparison results are significant, but the asterisk symbols (**) indicating significance are not shown in the graphs to avoid cluttering the figures.

**Fig. 7:**
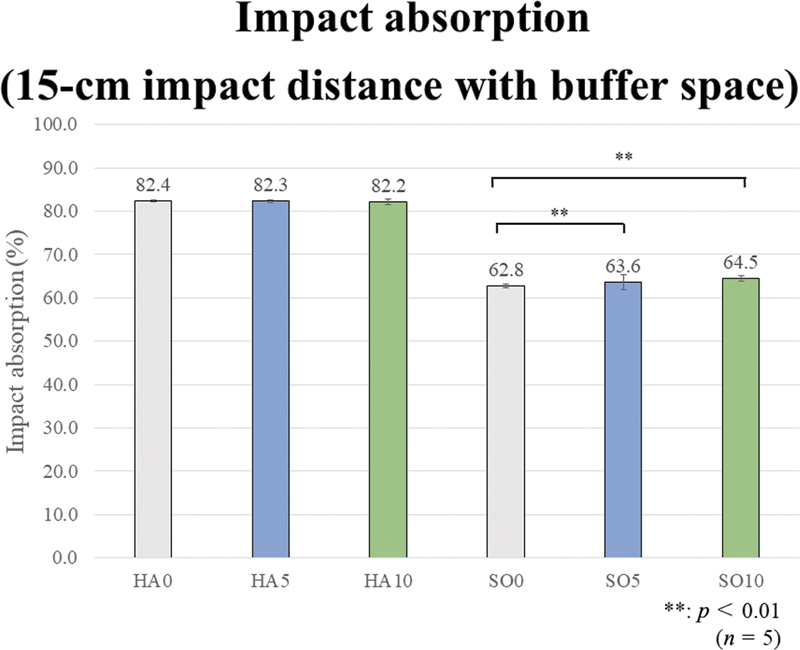
Impact absorption test results (15-cm impact distance with buffer space). Regarding the effect of ethylene vinyl acetate (EVA) material hardness, the impact absorption rates of the hard (HA) group are significantly higher than those of the soft (SO) group, regardless of the presence or added amount of glass fibers (GFs). Regarding the effect of fiber addition and content, no significant effect is observed in the HA group. In the SO group, SO5 and SO10 show significantly higher values than SO0.

**Fig. 8:**
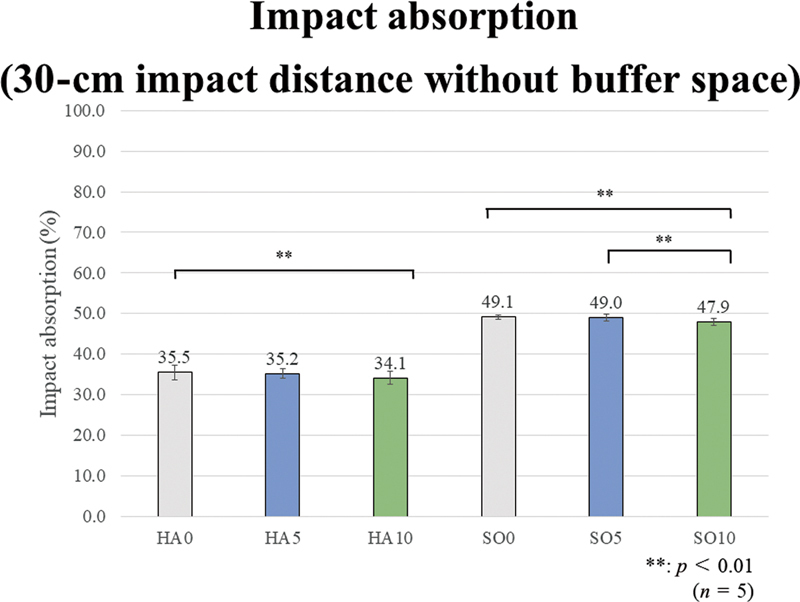
Impact absorption (30-cm impact distance without buffer space). Regarding the effect of ethylene vinyl acetate (EVA) material hardness, the impact absorption rates of the soft (SO) group are significantly higher than those of the hard (HA) group, regardless of the presence or added amount of glass fibers (GFs). Regarding the effect of fiber addition and content, in the HA group, HA10 shows significantly lower values than HA0. In the SO group, SO10 shows a significantly lower value than SO0 and SO5.

**Fig. 9:**
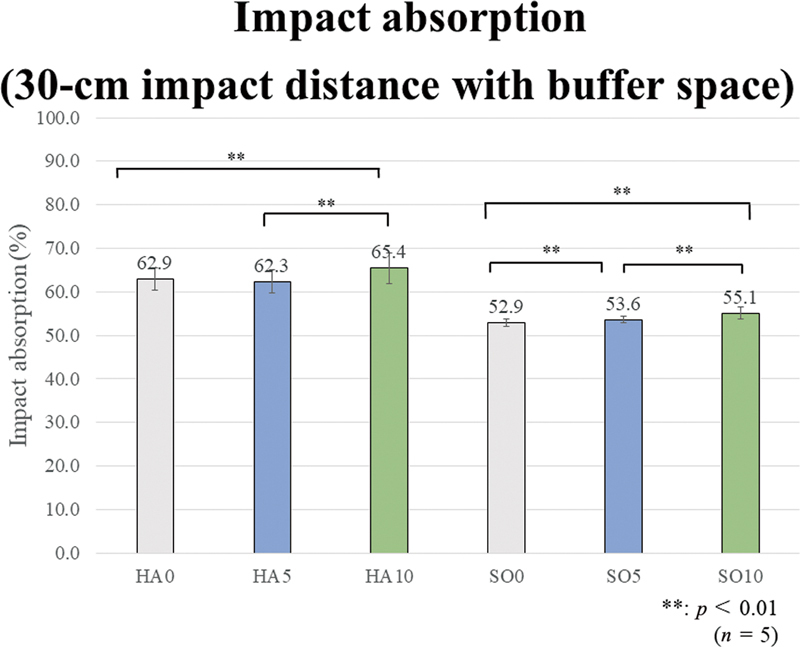
Impact absorption (30-cm impact distance with buffer space). Regarding the effect of fiber addition and content, in the hard (HA) group, HA10 shows a significantly higher value than HA0 and HA5. In the soft (SO) group, SO10 and SO5 show significantly higher values than SO0, and SO10 shows a significantly higher value than SO5.

**Table 5:** Impact absorption capacity (one-way ANOVA)

	Sum of squares	df	Mean square	*F*	*p*
**15-cm impact distance without buffer space**					
Factor	1.227	5	0.245	1,066.357	<0.01
Error	0.056	245	0		
Total	1.283	250			
**15-cm impact distance with buffer space**					
Factor	2.61	5	0.522	6,832.009	<0.01
Error	0.019	245	7.64E–05		
Total	2.629	250			
**30-cm impact distance without buffer space**					
Factor	0.724	5	0.145	283.997	<0.01
Error	0.125	245	0.001		
Total	0.849	250			
**30-cm impact distance with buffer space**					
Factor	1.417	5	0.283	1,339.262	<0.01
Error	0.054	245	0		
Total	1.471	250			

Abbreviation: ANOVA, analysis of variance.

The results for the 15-cm impact distance without the buffer space are as follows. In terms of the effect of EVA material hardness, the impact absorption rates of the SO group are significantly higher than those of the HA group, regardless of the presence or added amount of GFs. Regarding the effect of GF addition and content, in the HA group, no significant effect is observed. In the SO group, SO10 shows significantly lower values than SO0 and SO5.

The results for the 15-cm impact distance with a buffer space are as follows. Regarding the effect of EVA material hardness, the impact absorption rates of the HA group are significantly higher than those of the SO group, regardless of the presence or added amount of GFs. Regarding the effect of GF addition and content, no significant effect is observed in the HA group. In the SO group, SO5 and SO10 demonstrate significantly higher values than SO0.

The results for the 30-cm impact distance without a buffer space are as follows. As in the case with the 15-cm impact distance without a buffer space, regarding the effect of EVA material hardness, the impact absorption rates of the SO group are significantly higher than those of the HA group, regardless of the presence or added amount of GFs. Regarding the effect of GF addition and content, in the HA group, HA10 demonstrates a significantly lower value than HA0. In the SO group, SO10 has a significantly lower value than SO0 and SO5.

The results for the 30-cm impact distance with a buffer space are as follows. As in the case with the 15-cm impact distance with a buffer space, regarding the effect of EVA material hardness, the impact absorption rates of the HA group are significantly higher than those of the SO group, regardless of the presence or added amount of GFs. Regarding the effect of GF addition and content, in the HA group, HA10 demonstrates a significantly higher value than HA0 and HA5. In the SO group, SO5 and SO10 show significantly higher values than SO0, and SO10 has a significantly higher value than SO5.

**Fig. 10:**
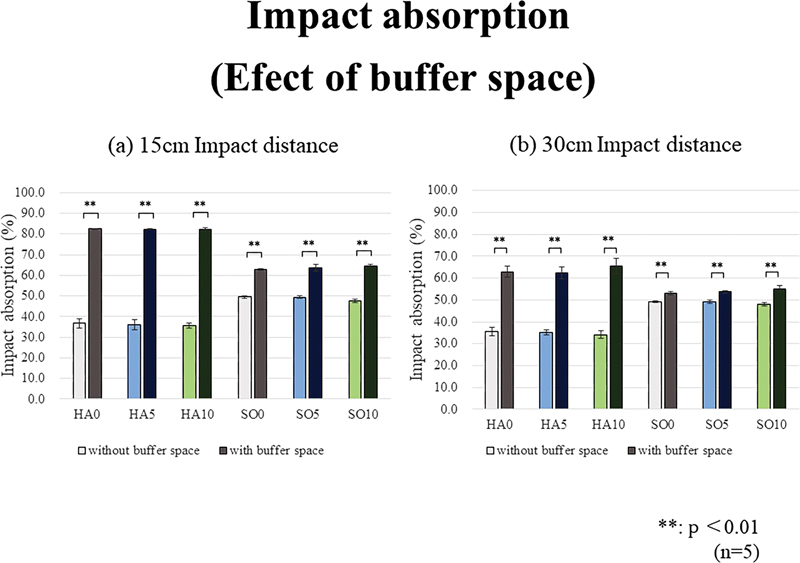
Regarding the presence or absence of buffer space, the impact absorption rate increases significantly with the addition of buffer space in all prototype materials under two test impact distance conditions.

Regarding the presence or absence of buffer space, the impact absorption rate increases significantly with the addition of such a space in all prototype materials under two test impact distance conditions ( Table 6 and Fig. 10 ).

**Table 6:** Effect of buffer space (Student's *t* -test)

Impact distance (cm)	Material name	Buffer space	Mean	SD	*p*
15	HA0	Without	0.367	0.023	< 0.01
		With	0.824	0.002	
	HA5	Without	0.36	0.023	< 0.01
		With	0.823	0.003	
	HA10	Without	0.355	0.012	< 0.01
		With	0.822	0.006	
	SO0	Without	0.494	0.006	< 0.01
		With	0.628	0.005	
	SO5	Without	0.494	0.008	< 0.01
		With	0.636	0.018	
	SO10	Without	0.476	0.008	< 0.01
		With	0.645	0.007	
30	HA0	Without	0.355	0.018	< 0.01
		With	0.629	0.026	
	HA5	Without	0.352	0.012	< 0.01
		With	0.623	0.027	
	HA10	Without	0.341	0.016	< 0.01
		With	0.654	0.035	
	SO0	Without	0.491	0.005	< 0.01
		With	0.529	0.008	
	SO5	Without	0.49	0.008	< 0.01
		With	0.536	0.007	
	SO10	Without	0.479	0.008	< 0.01
		With	0.552	0.014	

Abbreviation: SD, standard deviation.

## Discussion

The basic mechanical properties of EVA, namely, durometer hardness, Young's modulus, and tensile strength, were significantly higher in the HA group (low VA content) than in the SO group (high VA content), regardless of the presence or added amount of GFs. Moreover, the addition of GFs, especially in a large amount (10 wt%), significantly increased the above values. Moreover, impact absorption was significantly affected by the hardness of the original EVA material and significantly enhanced by the buffer space, regardless of the material and impact distance. In the presence of the buffer space, the addition of more GFs increased impact absorption. Interestingly, with the buffer space, the HA specimens exhibited significantly higher impact absorption capacities than the SO specimens; conversely, without the buffer space, the SO specimens had significantly higher impact absorption capacities than the HA specimens. That is, the hard EVA material was effective with the buffer space, and the soft EVA material was effective without a buffer space.

EVA, a random copolymer of ethylene and VA, is physiologically inert; it has no specific toxic effects on the human body. One of the purposes of copolymerizing VA is to reduce its crystallinity and improve its properties, such as transparency and flexibility. This decrease in crystallinity because of the introduction of VA governs the basic properties of copolymers. The flexibility, rubber elasticity, low-temperature properties, and other properties of EVA differ depending on the VA content (low percentage to ∼45%). 18 Because EVA has low crystallinity, it can maintain a certain strength after the blending of a large amount of filler. EVA is a thermoplastic resin that hardens when cooled and softens and flows again when the hardened resin is heated again. Even after being cooled and hardened, it can be molded again by applying heat. It has good moldability and suits mass production, and therefore it has low production cost and can be recycled. However, EVA might have certain disadvantages; for instance, its strength is inferior to that of thermoset resins, and it easily discolors. Therefore, resin additives are used for improvement. Resin additive effects include (1) stabilizers that prevent the deterioration of mechanical properties and color tones because of exposure to high temperatures during processing and ultraviolet exposure during use and (2) function-imparting agents that increase mechanical strength and control flexibility (reinforcer). 23 In this study, GFs were added to increase the mechanical strength of EVA. GFs are the first inorganic fibers produced for industrial use and are made by stretching molten glass. As dental materials, they are often used in combination with composite resins. They are applied to denture base materials. GFs have high tensile strength and elastic modulus, good dimensional stability, heat resistance, nonflammability, and established surface treatment technology that considers their adhesion to resins. 42

This study used EVA materials with different hardnesses (HA and SO) and GFs of different concentrations as reinforcing fibers to prepare the prototype materials. Results demonstrated that the mechanical properties of the prototypes, namely, durometer hardness, Young's modulus, and tensile strength, were significantly higher in the HA group than in the SO group. The specimens with GFs showed significantly better durometer hardness, tensile strength, and Young's modulus compared with the control SO and HA specimens. Moreover, the addition of GFs, especially in a large amount (10 wt%), significantly increased these values. These results agree with those in many previous studies. 29 30 31 32 33 34 44 45 46 47 A lower VA compounding ratio results in a higher Young's modulus and higher tensile strength. 25 The reinforcement effect of GFs works as follows. Compared with conventional polymer materials, GF-reinforced polymers have been successfully applied primarily because of their high specific modulus and strength. Because of the high modulus of elasticity of GFs, they receive most stresses without deforming. 34 Thus, GF reinforced specimens can exhibit improved characteristics. Furthermore, GF reinforcement significantly increases acrylic resin's flexural strength, impact strength, toughness, and Vickers hardness. 44 45 46 47 48 49 The integration of E-GFs significantly reduced the deformation of a denture base to less than 1%. 49 GFs improved the flexural and compression behavior 29 and flexural strength of heat-polymerized polymethyl methacrylate (PMMA) resin. 30 According to a previous study, 35 the hardness and flexural strength of a light-cured intermediate material (Innerframe LC) increased with the added amount of GFs. Consistent with the above-mentioned results, the addition of GFs, which have high tensile strength and elastic modulus, to EVA, a composite material similar to PMMA and the above intermediate material, improved the mechanical properties of the copolymer.

The placement of the buffer space led to high impact absorption capacities at any impact distance and for any specimen material. This space could prevent or reduce contact between the MG material's inner surface and the plastic plate's outer surface. The HA specimens with buffer spaces had high impact absorption capacities. The SO specimens without buffer spaces had high impact absorption capacities. This was because without the buffer space, the impact energy reduction depended on the absorption capacity of the MG material only; thus, more energy could be absorbed by the soft material (SO). However, with the buffer space, the hard material (HA) would not directly contact the acrylic plate or teeth up to a certain impact force or reduce the energy owing to deformation of the EVA MG material in the space. Consequently, the hard material (HA) with the buffer space resulted in a high impact absorption capacity. This result was similar to those for H&S MGs. 15 35 43 50 Further, the impact absorption capacity significantly increased with the amount of fibers added to HA; this enhancement was attributed to the increases in the EVA's hardness and Young's modulus caused by the large amount of added fibers. Considering EVA materials' differences in VA content, the addition of fibers, and the differences in the added amounts of fibers, better MG materials should be fabricated.

The properties required for MG materials differ depending on the MG part where they are used. On a maxillary incisor labial surface, where trauma is common, the teeth and alveolar bone should be protected. This area is possibly impacted directly from the front. If the inner layer of the MG is made of a harder material, some stresses occurring in the affected area can be reduced, and damage to the tooth surface can be limited. 36 Furthermore, the buffer space is essential. The MG material in this area should be hard enough to flex upon impact without contacting the teeth as much as possible. The material must be strong enough to avoid damage by impact. For improved MG fit and stability, the canine crown labial and anterior gingival portions should not have buffer spaces, and they should be made of soft, high-durability materials. Moreover, for protection of the lips, the outermost layer of the MG should be softer than the mucosal soft tissue. Occlusal surfaces, which must not have buffer spaces, must be able to withstand long-term pressure and absorb the impact energy transmitted from the mandible. In this area, high-strength materials are required to resist direct contact with the opposing teeth and high impact absorption. The palatal region should have as little as possible or no MG material to reduce discomfort during MG use. Therefore, sufficient strength on the buccal sides of the premolar and molars is required to maintain positional stability and prevent the MG from falling off.

Excellent MGs should be fabricated by changing the materials in each area, taking advantage of the difference in properties caused by variations in the amounts of VA and added fibers. Three-dimensional (3D) printing, a digital technology, is required to accurately accomplish this aim. This technology has evolved considerably, and it will be shortly applied to MGs. However, current lamination technology can be used at the moment. For the maxillary incisor labial surfaces, materials such as HA10 with buffer spaces should prevent or reduce injuries. This material, which demonstrated high tensile strength and Young's modulus values, suits other labial and buccal surfaces of the teeth. The material in the palatal region may be eliminated by ensuring sufficient retention using proper materials on the buccal side such as HA10. Regarding soft-tissue areas, it is difficult to provide a buffer space from the viewpoint of compatibility and retention, and therefore material such as SO10 is suitable. As for the occlusal surfaces, a buffer space cannot be added, and so material such as SO10 seems suitable. However, for strong clenching, two layers may have to be created using materials with different properties.

Only GFs were used as reinforcing fibers in this study. Other FRPs are available such as carbon FRPs. Carbon fibers are reportedly stronger than GFs. However, the high cost of this material should be addressed in future studies, and its safety for the human body should be explored. Regarding the application of 3D printing technology, 37 38 39 software issues, material development, adhesion, and accuracy should be considered. Moreover, deformation because of occlusal force, measurement, and changes in retention force should be analyzed. Moreover, deformation because of occlusal force, measurement, and changes in retention force should be analyzed. Long-term use of MGs may lead to deterioration and occlusal surface wear, potentially contributing to the protrusion of GFs and raising concerns about potential harm to periodontal tissues. Currently, dental materials are often reinforced with GFs. However, some reports have shown that GF-reinforced retainers can cause the accumulation of dental plaque and calculus. 51 Similar considerations may apply to EVA and, therefore, this should be considered as a subject for future investigation. Finally, researchers should consider GFs' heat resistance and noncombustibility because these are required for accurate occlusal adjustment using articulators or during setting, which requires heating by flame. Finally, researchers should consider GFs' heat resistance and noncombustibility because these are required for accurate occlusal adjustment using articulators or during setting, which requires heating by flame.

## Conclusion

The following conclusions were obtained from this study. The basic mechanical properties of EVA, namely, durometer hardness, Young's modulus, and tensile strength, were significantly higher in the HA group than in the SO group, regardless of the presence or added amount of GFs. The addition of GFs, especially in a large amount (10 wt%), increased these values significantly. The hardness of the original EVA material strongly affected impact absorption, and placement of the buffer space effectively enhanced impact absorption, regardless of the material and impact distance. Interestingly, the SO had a high shock absorption capacity without the buffer space. Moreover, the HA material had a high shock absorption capacity with the buffer space. Further, with the buffer space, a larger amount of added GFs increased impact absorption.
